# Parsimonious description for predicting high-dimensional dynamics

**DOI:** 10.1038/srep15736

**Published:** 2015-10-29

**Authors:** Yoshito Hirata, Tomoya Takeuchi, Shunsuke Horai, Hideyuki Suzuki, Kazuyuki Aihara

**Affiliations:** 1 Institute of Industrial Science, The University of Tokyo, 4-6-1 Komaba, Meguro-ku, Tokyo 153-8505, Japan; 2 Graduate School of Information Science and Technology, The University of Tokyo, Bunkyo-ku, Tokyo 113-8656, Japan; 3 CREST, JST, 4-1-8 Honcho, Kawaguchi, Saitama 332-0012, Japan

## Abstract

When we observe a system, we often cannot observe all its variables and may have some of its limited measurements. Under such a circumstance, delay coordinates, vectors made of successive measurements, are useful to reconstruct the states of the whole system. Although the method of delay coordinates is theoretically supported for high-dimensional dynamical systems, practically there is a limitation because the calculation for higher-dimensional delay coordinates becomes more expensive. Here, we propose a parsimonious description of virtually infinite-dimensional delay coordinates by evaluating their distances with exponentially decaying weights. This description enables us to predict the future values of the measurements faster because we can reuse the calculated distances, and more accurately because the description naturally reduces the bias of the classical delay coordinates toward the stable directions. We demonstrate the proposed method with toy models of the atmosphere and real datasets related to renewable energy.

Nonlinear time series analysis^1^,^2^,^3^, or time series analysis based on dynamical systems theory^4^,^5^,^6^, has been developed intensively in the last 35 years. The most important result is the method of delay coordinates^7^,^8^,^9^: Suppose that we can observe a scalar time series 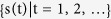 from a target system whose dimension is m. If we construct d-dimensional vectors 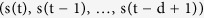, called delay coordinates, by using successive scalar measurements, then it is generally true that if d > 2m, the states x(t−d+1) for the underlying dynamical system and the vectors with delay coordinates 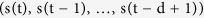, are one-to-one on the attractor, or a set of states the trajectory of the underlying dynamical system is attracted after the transient. Although this statement is supported how large the dimension m for the underlying dynamics is, practically the method of delay coordinates cannot be used for high-dimensional dynamics partly because the delay coordinates are distorted toward the stable directions on the attractor^10^, and partly because the calculation of delay coordinates becomes expensive.

The core part of the proposed method came from the idea of weighted delay coordinates^4^. In the weighted delay coordinates, the distortion of high-dimensional delay coordinates toward the stable directions is avoided by reducing weights of the past observations exponentially. If we denote the decay rate by λ (0 < λ ≤ 1), the weighted delay coordinates can be written as 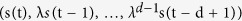. Berry *et al.*^10^ uses the weighted delay coordinates for dimension reduction.

Our idea is to virtually use infinite-dimensional weighted delay coordinates, namely 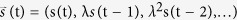, where we extend the time axis toward the minus infinity and assume s(t) = 0 for t ≤ 0 for simplicity. As we will show in the Methods section, the distance between 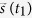 and 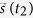 in 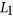 norm is calculated efficiently and successively. To make the distance converge, we just need to set 0 < λ < 1. Thus, by combining with the Lorenz’s method of analogues^11^,^2^, we construct a method of time series prediction (see the details in the Methods section).

First, we tested the proposed time series prediction on Lorenz’96 I model^12^,^13^, which is the minimum model of the atmosphere (see Supplementary Information for the details of the numerical experiment). When we compared the performance of the proposed method with that of the conventional 10-dimensional delay coordinates, we found that the proposed method tended to achieve the higher correlation coefficient (see Supplementary Information for the definition) between the prediction and the corresponding actual values up to 5 steps ahead (Fig. 1(a,c)). The proposed method was significantly better than the persistent prediction, where we let the current values be the prediction for the future values. For one of the 100 tested time series, we compared the speed of calculations. The proposed method only consumed 0.72 seconds, while the conventional 10-dimensional delay coordinates consumed 14.66 seconds. We used a laptop computer with Intel Core i7 CPU(3GHz) with 16.0GB memory. The programs were implemented in MATLAB. We did not use the recursive formula of equation (4) in Methods here. Thus, the proposed method is faster and more accurate in prediction than the conventional delay coordinates.

Second, we tested the proposed time series prediction on Lorenz’96 II model^12^,^13^. In the Lorenz’96 II model, there are two types of variables: the slow variables correspond to the upper-layer of the atmosphere and the fast variables correspond to the layer close to the surface of the earth (see Supplementary Information for the details of this numerical experiment). We found that the proposed method tended to have the greater correlation coefficient than the conventional 10-dimensional delay coordinates up to 10 steps ahead (Fig. 1(b,d)). In one of the 100 tested time series, the computational time required for the proposed method was 0.80 seconds with the laptop computer, while that required for the conventional delay coordinates was 20.16 seconds; we did not use the recursive formula of equation (4) here too.

Third, we applied the proposed time series prediction to the sunshine duration at a single point of Fuchu, Japan (see Supplementary Information for the details of the numerical experiment). The result presented in Fig. 2 shows that the proposed method achieved the higher correlation coefficient than the persistent prediction and the prediction using 1 day periodicity when the prediction steps were greater than or equal to 1.2 hours. To make the prediction for the 10 minutes dataset spanning 5 years of 2008–2012, it only took 3.8 hours with a desktop computer with 2.7 GHz 12-Core Intel Xenon E5 with 64 GB memory. Therefore, the proposed method can run in the real time.

We also applied the proposed time series prediction to the wind speed data at Fuchu, Japan (See the Supplementary Information for the details of this numerical experiment). We found that the proposed method achieved the greater correlation coefficient than the persistent prediction and the prediction using 1 day periodicity when the prediction steps were between 4 and 15.8 hours (Fig. S1). In addition, we needed 3.8 hours with the desktop computer to complete the prediction for the dataset between 2008 and 2012. Thus, for this wind speed dataset, the prediction can also be done in the real time.

By using the proposed method, we can circumvent problems of the current standard practice for obtaining delay coordinates^2^, which is, for example, to decide the delay by the first minimum of mutual information^14^ and the embedding dimension by false nearest neighbors^15^. Instead of choosing these two parameters, we need to choose the decay rate λ, by which the proposed method shows the robust performance as demonstrated in Fig. 3 and S2. Thus, the proposed method will make it easier to apply nonlinear time series prediction. We can even remove the choice of λ by combing the proposed method with the expert advice algorithm^16^,^17^,^18^ (see Supplementary Information for the details). Thus, the proposed method is suitable for automating some time series prediction tasks.

The proposed method is robust against the observational noise. Even if we increase the noise level up to 10% of the standard deviation of the original time series, the correlation coefficient between the prediction using the proposed method and the actual values was significantly higher than that between the prediction using the conventional delay coordinates and the actual values (See Fig. 4). The proposed method naturally filters out observational noise when it is included in the measurements. Moreover, even if we evaluate the prediction with the root mean square errors, the proposed method is superior to the conventional delay coordinates for short-term predictions (Fig. 1(e,f)). Thus, our results could have the robustness to some extent in terms of ways for evaluating the prediction.

If we increased the size of database, then the prediction performance became better (Fig. S3). In addition, even if we used different numbers of neighbors for making the prediction, the performance was robust and did not change much (Fig. S4).

The lower the minimal Lyapunov exponent *σ*_1_ for the underlying dynamics is, the higher the optimal λ is (Fig. S5). But, judging from the values of the optimal λ for the prediction, the reconstructed space was not reduced to the most stable direction because 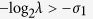
10. Therefore, the list of distances contained the information of more than the one-dimensional space. From this viewpoint, the proposed method provides a convenient description for the high-dimensional dynamics.

It is easy to further extend the proposed infinite-dimensional weighted delay coordinates to multivariate time series^19^,^20^,^21^,^22^,^23^,^24^ or point processes^25^,^26^,^27^,^28^,^29^. Let *W* be a set of states. If we define the state at time t by 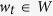 and a distance function on these states by 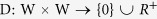, then the distance 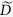 between 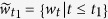 and 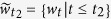 on such infinite dimensional weighted delay coordinates can be defined as


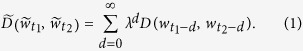


This distance, called the Fréchet product metric^30^, satisfies the three conditions for the metric: (i) 
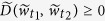
 and 
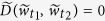
 if and only if 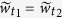; (ii) 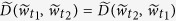; (iii) 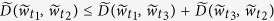. Therefore, if a given time series is multidimensional, we may choose the Euclidean distance between times 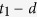 and 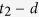 as *D* to obtain 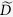. We will discuss this extension in our future communication.

The proposed description might also be useful in inferring a network structure. This is an open question and we are also interested in developing the method in this direction.

Comparing with the traditional delay coordinates, the proposed infinite-dimensional weighted delay coordinates can produce more accurate time series prediction faster. As there is an increasing demand for real-time prediction for a big dataset especially in the field of renewable energy such as photovoltaic and wind powers, we hope that the proposed method helps to introduce more renewable energy into the power grids so that we can reduce CO_2_ emissions.

## Methods

Suppose that a scalar time series 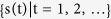 is given successively. Denote by N the size of the database. Let 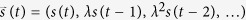 be the infinite-dimensional weighted delay coordinates, where λ is a decay rate. We define a distance 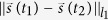 between 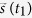 and 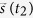 as


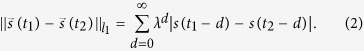


We use λ = 0.5 if not mentioned.

This distance is convenient because we can reuse the previous calculations of distances to obtain distances for a pair of its future infinite-dimensional weighted delay coordinates. Namely, observe the following relation:


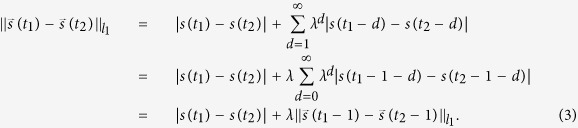


When we use the conventional *d*-dimensional delay coordinates (λ = 1), the similar recursive logic may be applied to simplify the calculation of distances as follows:


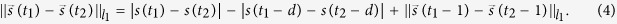


We combine the relation of equation (3) with Lorenz’s method of analogues^2^,^11^. In the Lorenz’s method of analogues, we find close matches in the past and average their following points as prediction for the future. Let us construct prediction of up to P steps ahead. Suppose that the current time is at time t and we have the following datasets: a list of distances 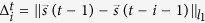 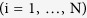 and a list of the corresponding observed values 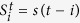 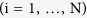. First, we find the set 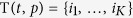 of indices for the K smallest distances among 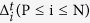, and provide the p steps ahead prediction for each p 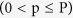 by 
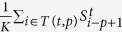
. We used K = 10 if not mentioned. Second, we update the list of distances by


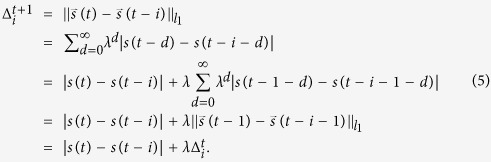


We also update the list of the corresponding values by


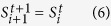


for 1 ≤ i < N and


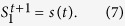


Therefore, to run the proposed method, each time we obtain a measurement, we only need to store 2N floating numbers, compare N floating numbers, conduct N additions, N subtractions, and N multiplications. Compare these numbers with the case using the traditional d-dimensional delay coordinates: If we do not use the recursive formula of equation (4), we need dN comparisons for comparing element-wisely *N* pairs of *d* dimensional delay coordinates, dN subtractions, and (d−1)N additions (The computation for finding nearest neighbors is in the same order).

## Additional Information

**How to cite this article**: Hirata, Y. *et al.* Parsimonious description for predicting high-dimensional dynamics. *Sci. Rep.* **5**, 15736; doi: 10.1038/srep15736 (2015).

## Supplementary Material

Supplementary Information

## Figures and Tables

**Figure 1:**
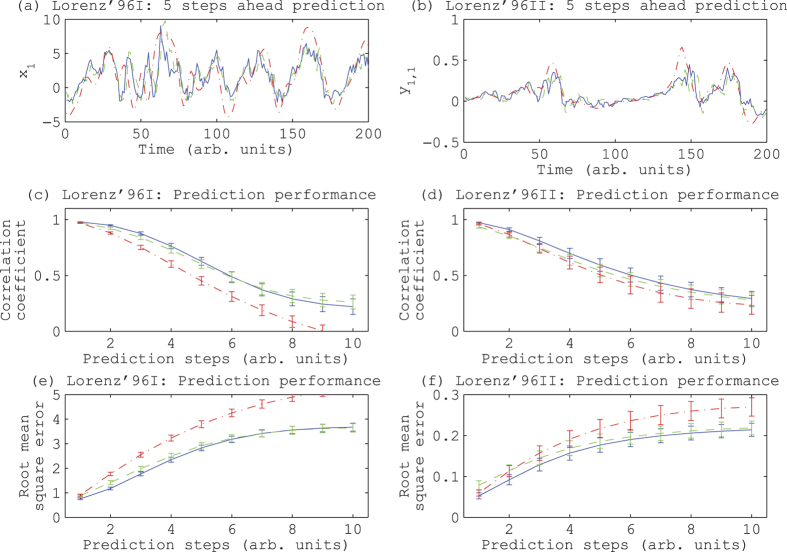
Prediction results on Lorenz’96 I model and Lorenz’96 II model. (**a**,**c**,**e**) correspond to the Lorenz’96 I model, while (**b**,**d**,**f**) correspond to the Lorenz’96 II model. (**a**) and (**b**) show examples of 5 steps ahead prediction, (**c**,**d**) show the correlation coefficients between the prediction and the actual value given a prediction step, and (**e**,**f**) show the root mean square errors given a prediction step. In panels (**a**,**b**), the blue solid line corresponds to the prediction by the proposed infinite-dimensional weighted delay coordinates, the green dashed line corresponds to the one by the 10-dimensional delay coordinates, and the red dash-dotted line, the actual values. In panels (**c**–**f**), the blue solid line and the green dashed line correspond to the proposed method and the conventional 10-dimensional delay coordinates, respectively, and the red dash-dotted line, the persistence prediction. The error bars show the standard deviations.

**Figure 2:**
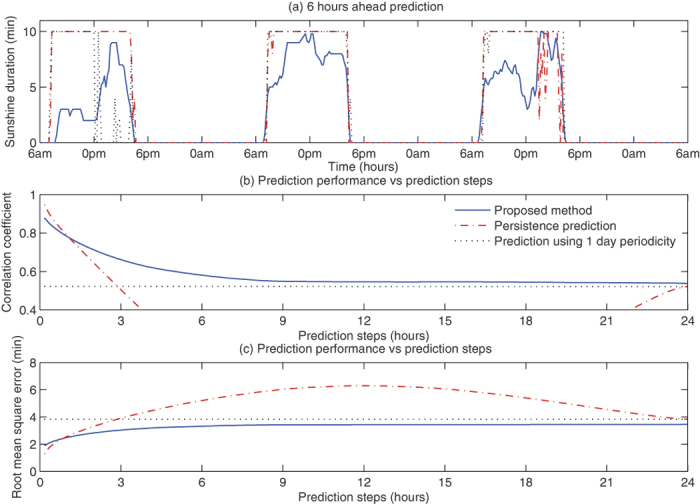
The prediction result on sunshine duration within 10 minutes at Fuchu, Japan. Panel (**a**) shows 6 hours ahead prediction (blue solid line), prediction using 1 day periodicity (black dotted line), and the actual values (red dash-dotted line). Panel (**b**) shows the correlation coefficients between the prediction and the actual observations. Panel (**c**) shows the root mean square errors between the prediction and actual observation. In panels (**b**,**c**), the blue solid line, the red dash-dotted line, and the black dotted line correspond to the proposed method, the persistence prediction, and the prediction using 1 day periodicity.

**Figure 3:**
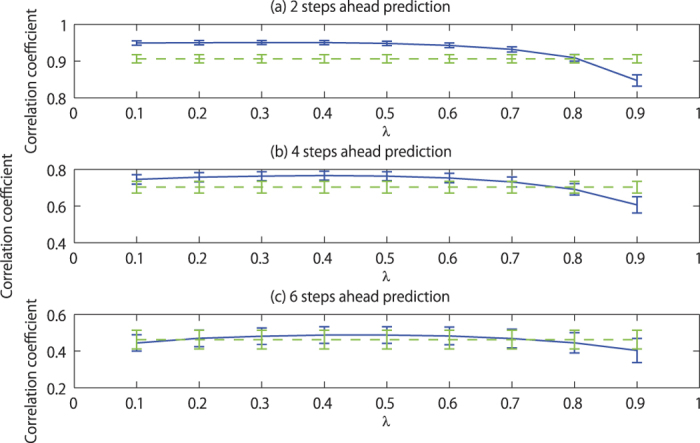
Dependence of the prediction performance on the parameter λ of the proposed method for Lorenz’96 I model. Panels (**a**–**c**) correspond to 2, 4, and 6 steps ahead predictions. In each panel, the blue solid line corresponds to the proposed method and the green dashed line corresponds to the conventional 10-dimensional delay coordinates, which do not depend on the choice of the parameter λ. Each error bar shows the standard deviation of the correlation coefficient between the prediction and the actual values.

**Figure 4:**
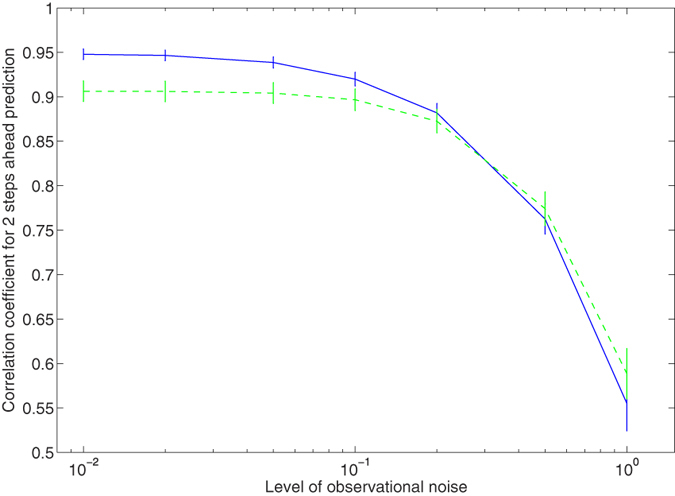
Dependence of the prediction performance on the level of the observational noise for Lorenz’96 I model. The blue solid line and the green dashed line correspond to the proposed method and the conventional 10 dimensional delay coordinates, respectively. The error bars show the standard deviations for the correlation coefficients between the prediction and the actual values.
