# Supplementary material for: Parsimonious description for predicting high-dimensional dynamics
Source: Sci Rep. 2015 Oct 29;5:15736. doi: 10.1038/srep15736 (PMC4625180; doi:10.1038/srep15736)
Supplement: Supplementary Information [file srep15736-s1.pdf]

Supplementary Information

Parsimonious description for predicting high-dimensional dynamics

Yoshito Hirata, Tomoya Takeuchi, Shunsuke Horai, Hideyuki Suzuki & Kazuyuki Aihara

Supplementary Methods

Supplementary Figs. 1-11

Supplementary Methods

Details of numerical experiments

Lorenz'96 I model

The Lorenz'96 I model[12,13] is defined as follows: We have

$$\dot{x}_i = -x_{i-2}x_{i-1} + x_{i-1}x_{i+1} - x_i + F, \qquad \text{(S1)}$$

$$x_i = x_{i+m}, \qquad \text{(S2)}$$

for each $i \in \{1,2,\dots,m\}$. We used $m = 10$ and $F = 8$ if not mentioned. We generated a time series of length 4000 sampling x_1 every 0.05 unit times if not mentioned after throwing away the transient parts. We used the first 2000 points as the database and the following 2000 points for testing predictions. Namely, we set $N = 2000$. The prediction steps were between 1 and 10 steps. To obtain Figs. 1(a), 1(c), 1(e), 3, 4, S3, and S4, we generated 100 time series to obtain the error bars. We generated 1000 time series by generating different F using the normal distribution of mean 8 and standard deviation 1 to obtain Fig. S5. In Fig. S5, we used the method of Kantz[31] backward in time to obtain the minimal Lyapunov exponent.

Lorenz'96 II model

The Lorenz'96 II model[12,13] is defined as follows: We have

$$\dot{x}_i = -x_{i-2}x_{i-1} + x_{i-1}x_{i+1} - x_i + F - \frac{h_x c}{b}\sum_j y_{j,i}, \quad \text{(S3)}$$

$$\dot{y}_{j,i} = -cby_{j+1,i}y_{j+2,i} + cby_{j-1,i}y_{j+1,i} - cy_{j,i} + \frac{h_y c}{b}x_i, \quad \text{(S4)}$$

$$x_i = x_{i+m}, \quad \text{(S5)}$$

$$y_{j+n,i} = y_{j,i+1}, \quad \text{(S6)}$$

for each $i \in \{1,2,\dots,\mathrm{m}\}$ and $j \in \{1,2,\dots,\mathrm{n}\}$, where we set $\mathrm{F} = 8, \mathrm{b} = 10, \mathrm{c} = 10, h_x = 1, h_y = 1, m = 40,$ and $\mathrm{n} = 5$. The variables x_i correspond to the slow variables over the sky and the variables $y_{j,i}$ correspond to the fast variables close the surface of the earth. We generated 100 time series of length 4000 with different initial conditions by observing $y_{1,1}$ every 0.01 unit times after removing the transient. For each time series, we used the first 2000 points as the database and the following 2000 points to test the predictions, i.e., $\mathrm{N} = 2000$. The prediction steps were between 1 and 10 steps ahead.

Sunshine duration data and wind speed data

The datasets were provided by the Japan Meteorological Agency. Both datasets span the time period between 1 January 2002 and 31 December 2012. We had measurements of sunshine duration and wind speed within 10 minutes at Fuchu, Japan, every 10 minutes. We set $\mathrm{N} = 6\times24\times(365\times6 + 2)$ to construct the database based on the dataset of years 2002-2007 and predicted the dataset of years 2008-2012. Namely, we used the

datasets of the first 6 years to predict the following 5 years. The prediction steps were between 10 and 1440 minutes. We used $\lambda = 0.5^{\frac{1}{6\times 24}}$.

Evaluation of prediction

We evaluate the prediction by using two indices. The first index is the correlation coefficient defined as

$$\frac{\sum_t (s(t)-\bar{s})(\hat{s}(t)-\bar{\hat{s}})}{\sqrt{\sum_t (s(t)-\bar{s})^2}\sqrt{\sum_t (\hat{s}(t)-\bar{\hat{s}})^2}}, \qquad \text{(S7)}$$

where $s(t)$ is the actual observation at time t, $\hat{s}(t)$ is its prediction, and $\bar{s}$ and $\bar{\hat{s}}$ are the means for the actual observation and the prediction, respectively.

The second index is the root mean square error defined as

$$\sqrt{\frac{1}{T_2-T_1}\sum_{t=T_1+1}^{T_2}(s(t)-\hat{s}(t))^2}, \qquad \text{(S8)}$$

where T_1 and T_2 define the time period when the prediction is evaluated.

Combining the proposed method with expert advice for automatically choosing appropriate λ

In the examples of sunshine duration and wind speed, we also attempted to choose the parameter λ automatically. For this sake, we combine the proposed method with

expert advice[16-18], especially one[17] with an exponential discounting.

We combine the proposed method and expert advice with the exponential discounting[17] in the following way. Let λ_l be the lth decay rate. Here we set $2^{-0.5^{0.25l}}$ $(l = 0,1,\dots,L-1)$ and $L = 37$. Let $\hat{s}_l^p(t)$ be the p steps ahead prediction by using the lth decay rate for time t. We also denote, by $v_l^p(t)$, the weight for the p steps ahead prediction at time t using the lth decay rate. We set $v_l^p(0) = 1/L$ for all p and l at the beginning. In addition, denote, by $e_l^p(t)$, the accumulated prediction error for the p steps ahead prediction using the lth decay rate at time t. We initially define $e_l^p(0) = 0$ for all p and l.

At time t, we apply the following algorithm. First, we evaluate $\hat{s}_l^p(t+p-1)$ using the proposed method for each $p \in \{1,2,\dots,P\}$ and each $l \in \{0,1,\dots,L-1\}$ and obtain the ensemble prediction

$$\sum_{l=0}^{L-1} v_l^p(t)\hat{s}_l^p(t+p-1) \qquad \text{(S9)}$$

for the prediction for time $(t+p-1)$, where $p \in \{1,2,\dots,P\}$. Second, we obtain a measurement $s(t)$ at time t and evaluate the accumulated prediction error with an exponential discounting $\alpha < 1$ as follows:

$$e_l^p(t) = \alpha e_l^p(t-1) + |s(t) - \hat{s}_l^p(t)| \qquad \text{(S10)}$$

for each $p \in \{1,2,\dots,P\}$ and $l \in \{0,1,\dots,L-1\}$. Third, we update the weights by

$$v_l^p(t) = \frac{exp(-\eta e_l^p(t))}{\sum_{l'} exp(-\eta e_{l'}^p(t))} \qquad \text{(S11)}$$

for each $p \in \{1,2,\dots,P\}$ and $l \in \{0,1,\dots,L-1\}$. Throughout the manuscript, we set $\alpha = 0.9$ and $\eta = 10$ if not mentioned.

The combination was tested using the datasets of sunshine duration (Fig. S6) and wind speed (Fig. S7). Each of these figures shows that the prediction can be improved by combining the proposed method and the expert advice with the exponential discounting. The weights for each dataset at the end of the algorithm are shown in Fig. S8 and Fig. S9, respectively. The weights for the sunshine duration were widely distributed (Fig. S8), while the weights for the wind speed were concentrated on a small number of decay rates (Fig. S9).

In this extension with expert advice, we introduced two hyper-parameters α and η to remove the effects of different λs. But, the proposed extension is not so much sensitive on the selections of these hyper-parameters α and η (see Figs. S10-S11). Therefore, including the two hyper-parameters is not so much a serious problem when we use this

extension.

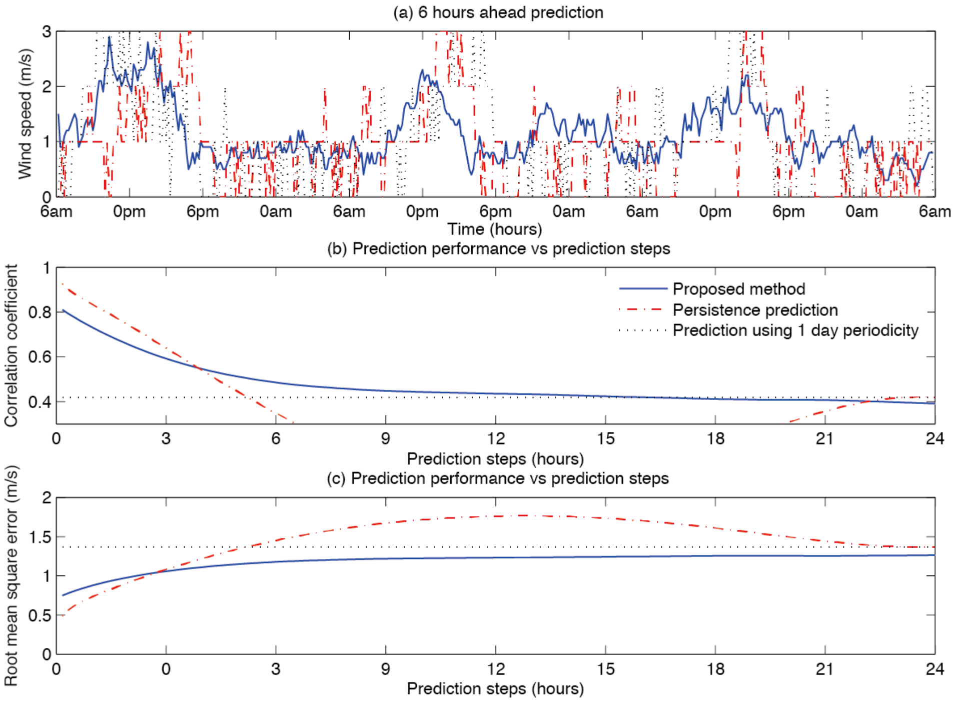

Figure S1 | Prediction results on wind speed data. Panel (a) shows 6 hours ahead prediction by the proposed method (blue solid line), the prediction using 1 day periodicity (black dotted line), and the actual observations (red dash-dotted line). Panel (b) shows the correlation coefficients between the prediction and the actual observations. Panel (c) shows the root mean square errors for the corresponding predictions. In panels (b) and (c), the proposed method, the persistence prediction, and the prediction using 1 day periodicity are shown in the blue solid line, the red dash-dotted line, and the black dotted line, respectively.

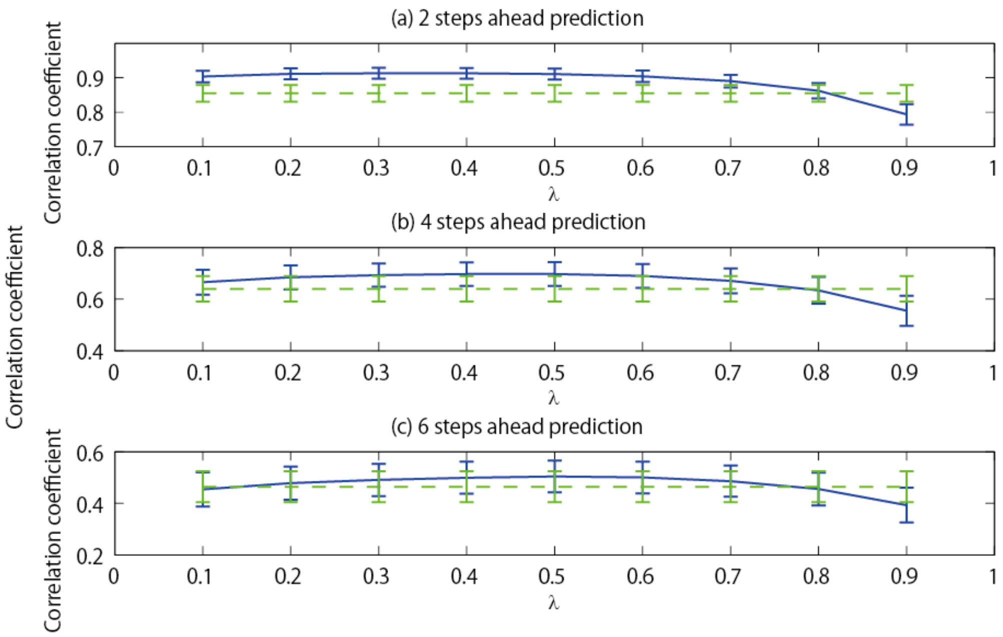

Figure S2 | Dependence of the prediction performance on the parameter λ of the proposed method for Lorenz'96 II model. See the caption of Fig. 3 to interpret the results.

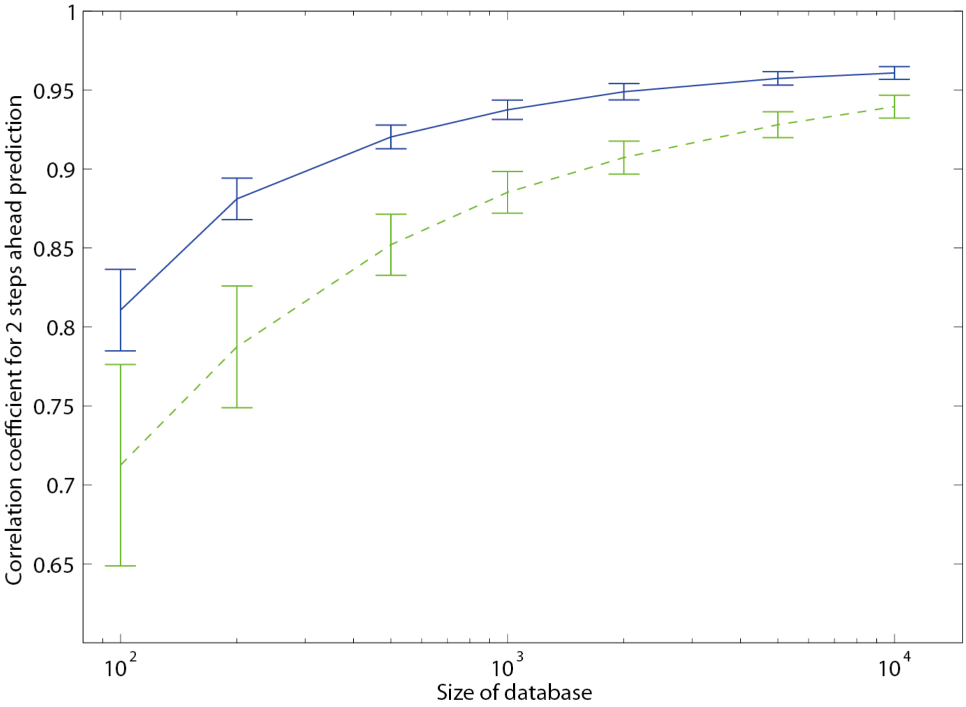

Figure S3 | Dependence of prediction performance on the size of database for the Lorenz'96 I model. See the caption of Fig. 4 to interpret the results.

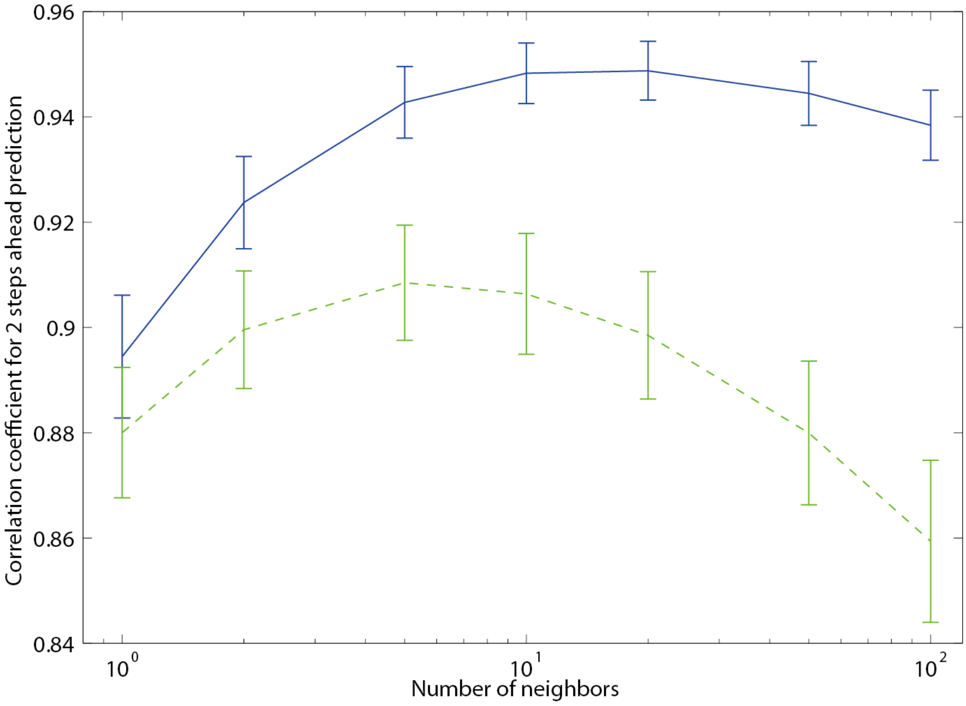

Figure S4 | Dependence of prediction performance on the number of neighbors used for prediction, Lorenz'96 I model. See the caption of Fig. 4 to interpret the results.

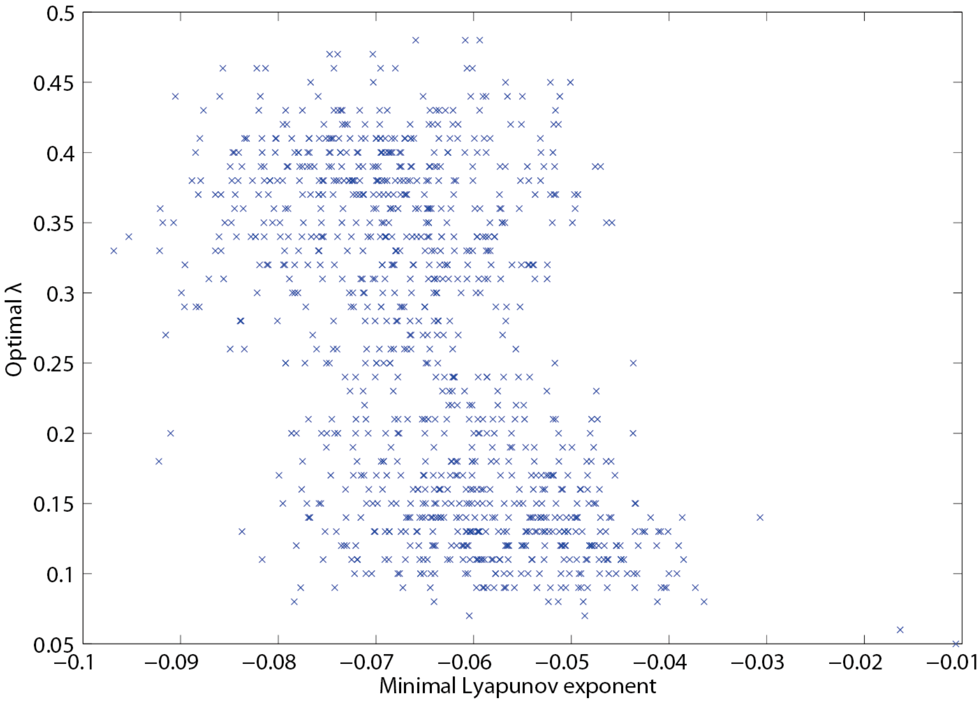

Figure S5 | Relation between the minimal Lyapunov exponent and the optimal parameter λ for the proposed method. The optimal λ was chosen by the grid search in the resolution of 0.01 so that we maximized the correlation coefficient between 2 steps ahead prediction by the proposed method and the actual values.

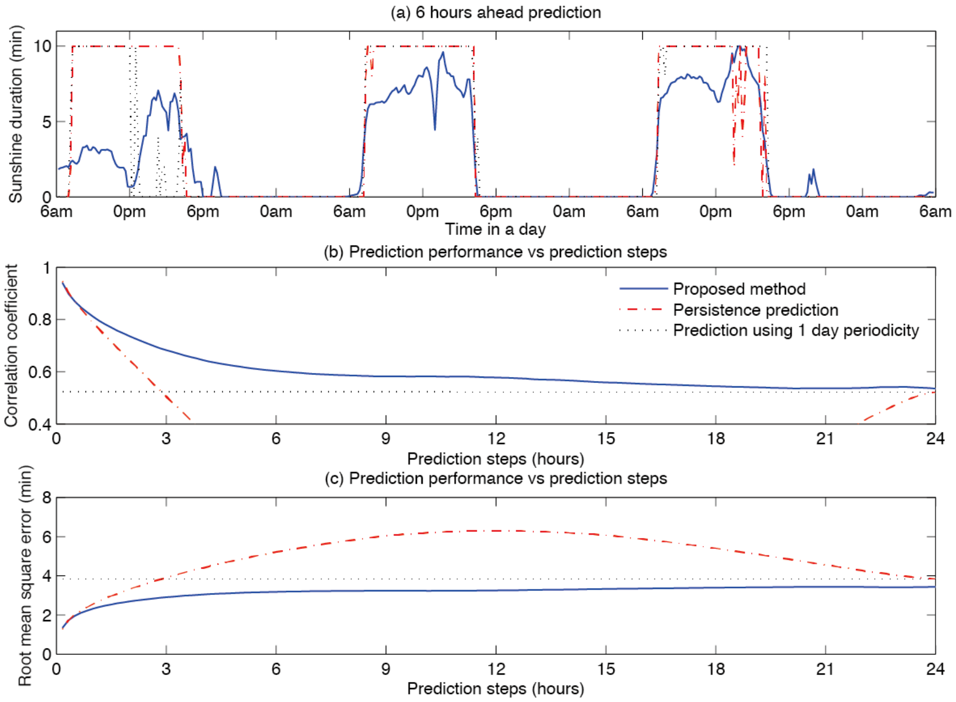

Figure S6 | Prediction results for the combination of the proposed method with expert advice on the dataset of sunshine duration. See Fig. 2 to interpret the results.

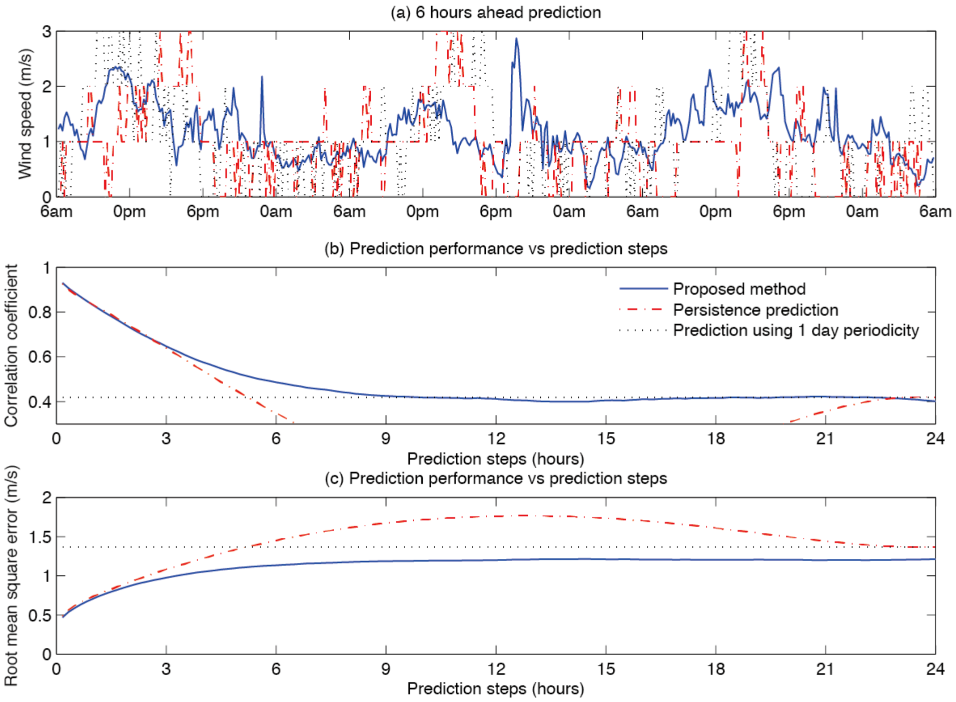

Figure S7 | Prediction results for the combination of the proposed mehod with expert advice on the dataset of wind speed. See Fig. S1 to interpret the results.

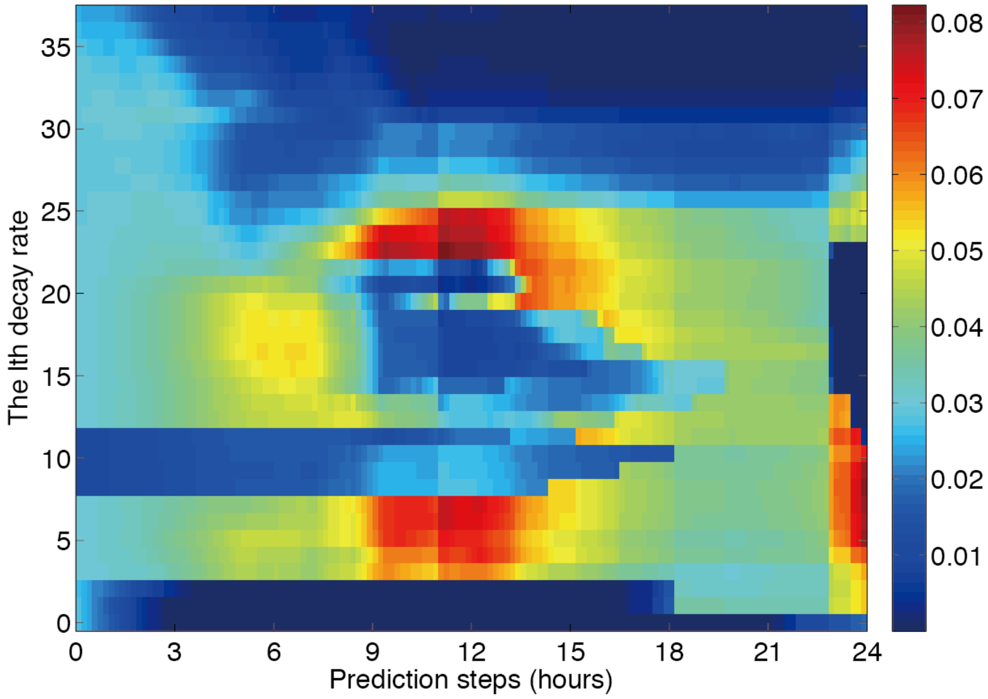

Figure S8 | Distribution for weights for the combination of the proposed method with expert advice on the dataset of sunshine duration.

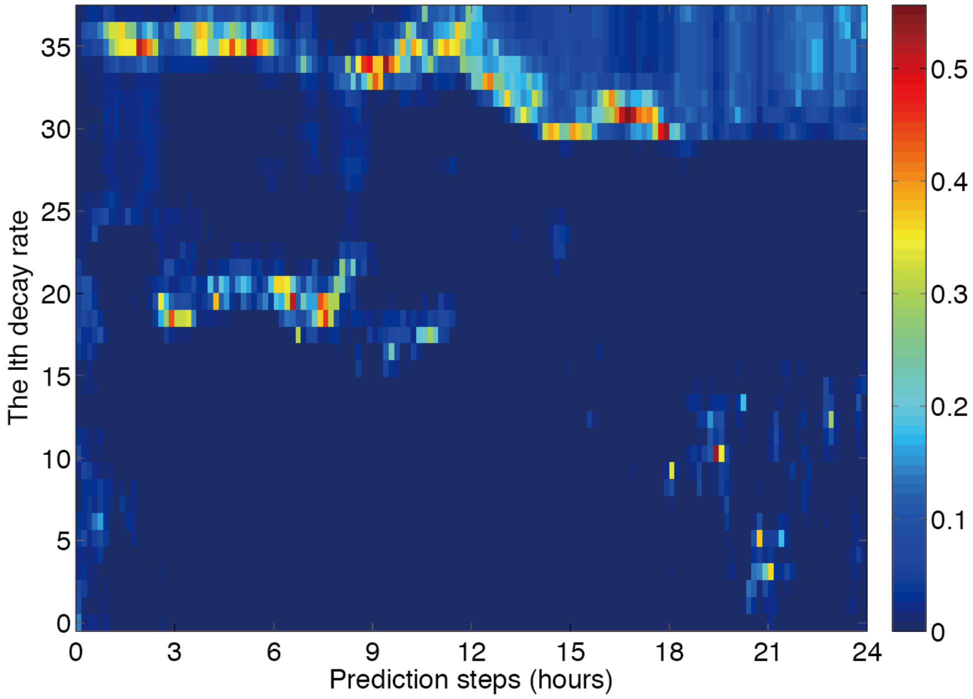

Figure S9 | Distribution for weights for the combination of the proposed method with expert advice on the dataset of wind speed.

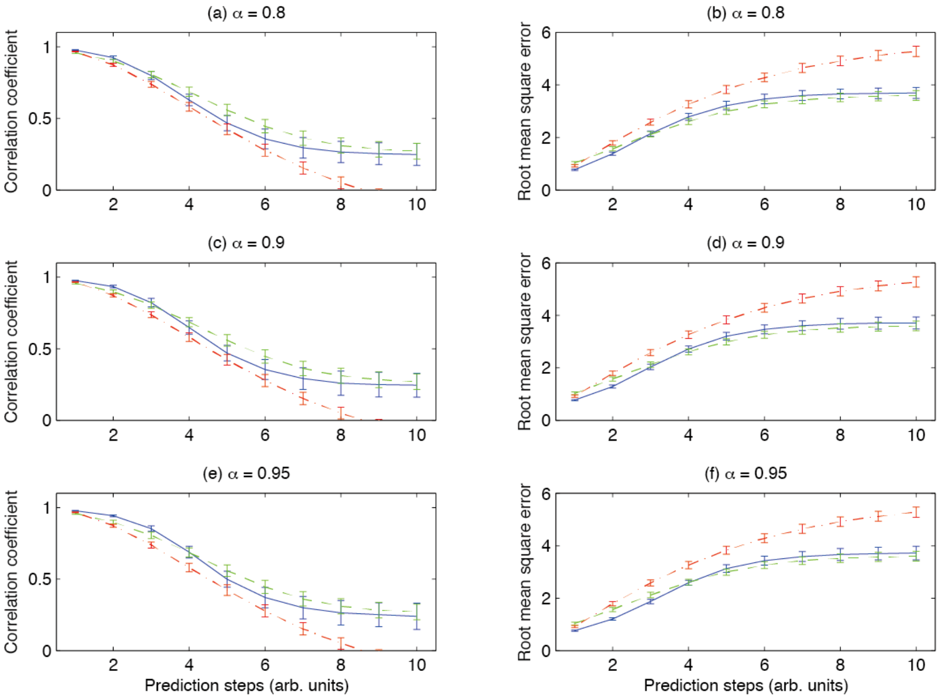

Figure S10 | Dependence of the proposed method with expert advice with hyper-parameter α on Lorenz'96 I model. Panels (a), (c), and (e) show the correlation coefficients given prediction steps, and panels (b), (d), and (f) show the root mean square errors given prediction steps. (a) and (b) correspond to $\alpha = 0.8$, and (c) and (d) correspond to $\alpha = 0.9$, and (e) and (f) correspond to $\alpha = 0.95$. The other hyper-parameter η was set to 10. The error bars show the mean and standard deviation obtained from 10 trials.

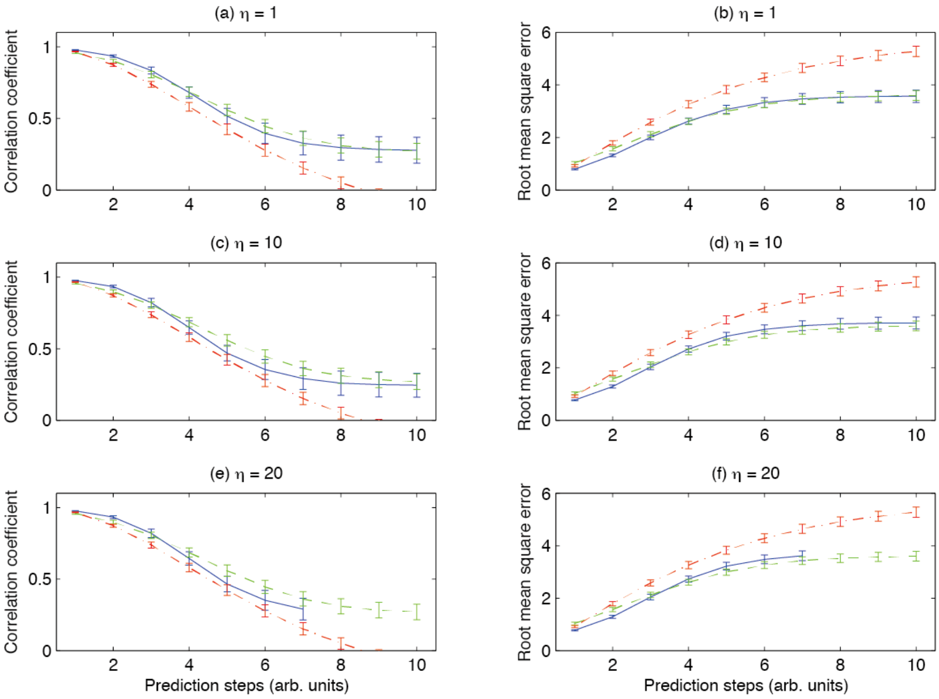

Figure S11 | Dependence of the proposed method with expert advice on the hyper-parameter η. (a), (c), and (e) show the correlation coefficients and (b), (d), and (f) show the root mean square errors. In panels (a) and (b), we set $\eta = 1$, in panels (c) and (d), we set $\eta = 10$, and in panels (e) and (f), we set $\eta = 20$. We set the other hyper-parameter $\alpha = 0.9$. The error bars show the mean and the standard deviation obtained over 10 trials. In panels (e) and (f), we could not calculate the error bars correctly because the weights become NaN when the prediction steps were more than 7.
